# Active travel to schools in Wales

**DOI:** 10.23889/ijpds.v8i2.2312

**Published:** 2023-09-14

**Authors:** Samantha Turner, Richard Fry, Lucy Griffiths, Jane Lyons, Rhodri Johnson, Amy Mizen, Ronan Lyons

**Affiliations:** 1 Swansea University, Swansea, United Kingdom

## Objectives

Active travel to school (ATS), such as walking and cycling, not only reduces carbon emissions and air pollution but also contributes to a myriad of health benefits. Understanding the ‘potential’ for ATS across Wales is poorly understood yet vital to inform policy and practice aimed at increasing ATS.

## Methods

Using geospatial techniques, network distances have been calculated for all residences to all schools in Wales. Distances for each residence were de-identified and imported into the Secure Anonymised Information Linkage (SAIL) databank using SAIL’s split file approach. Within SAIL, anonymised distances were linked to encrypted school locations and a cohort of approximately 440,000 children attending a state school in Wales as recorded in the Education Wales (EDUW) dataset. Travel distance to school was subsequently filtered for each child within our cohort.

## Results

The incorporation of geospatial network derived origin-destination pairs (n=2,205,000,000) allows us to explore how households can engage in ATS, and how this varies by primary/secondary school, welsh-speaking, urban/rural, and socioeconomic status. Initially we will present findings based on distance alone, with future refinements to encompass other factors influencing ATS including: active travel routes, road safety, and topography. We will link our results to anonymised ATS survey responses in SAIL, to calculate specific ATS distance thresholds by age, deprivation, and urban/rural status, including social indicators such as household composition.

## Conclusion

Currently there is limited local or national comparable information on the potential for ATS in Wales. This baseline information is urgently needed to inform ATS policy and planning, and to ensure appropriate ATS interventions are prioritised for schools and communities where need is greatest.

